# Antidiabetic Effect of Oral Borapetol B Compound, Isolated from the Plant *Tinospora crispa*, by Stimulating Insulin Release

**DOI:** 10.1155/2013/727602

**Published:** 2013-11-10

**Authors:** Faradianna E. Lokman, Harvest F. Gu, Wan Nazaimoon Wan Mohamud, Mashitah M. Yusoff, Keh Leong Chia, Claes-Göran Östenson

**Affiliations:** ^1^Department of Molecular Medicine and Surgery, Karolinska Institutet, Karolinska University Hospital, 171 76 Stockholm, Sweden; ^2^Department of Diabetes, Cardiovascular, Diabetes and Nutrition Research Centre, Institute for Medical Research, 50588 Jalan Pahang, Kuala Lumpur, Malaysia; ^3^Faculty of Industrial Sciences and Technology, Universiti Malaysia Pahang, Lebuhraya Tun Razak, 26300 Gambang, Pahang, Malaysia

## Abstract

*Aims*. To evaluate the antidiabetic properties of borapetol B known as compound 1 (C1) isolated from *Tinospora crispa* in normoglycemic control Wistar (W) and spontaneously type 2 diabetic Goto-Kakizaki (GK) rats. *Methods*. The effect of C1 on blood glucose and plasma insulin was assessed by an oral glucose tolerance test. The effect of C1 on insulin secretion was assessed by batch incubation and perifusion experiments using isolated pancreatic islets. *Results*. An acute oral administration of C1 improved blood glucose levels in treated versus placebo groups with areas under glucose curves 0–120 min being 72 ± 17 versus 344 ± 10 mmol/L (*P* < 0.001) and 492 ± 63 versus 862 ± 55 mmol/L (*P* < 0.01) in W and GK rats, respectively. Plasma insulin levels were increased by 2-fold in treated W and GK rats versus placebo group at 30 min (*P* < 0.05). C1 dose-dependently increased insulin secretion from W and GK isolated islets at 3.3 mM and 16.7 mM glucose. The perifusions of isolated islets indicated that C1 did not cause leakage of insulin by damaging islet beta cells (*P* < 0.001). *Conclusion*. This study provides evidence that borapetol B (C1) has antidiabetic properties mainly due to its stimulation of insulin release.

## 1. Introduction

Type 2 diabetes mellitus is a heterogeneous disorder associated with impaired insulin secretion from pancreatic **β**-cells and decreased insulin sensitivity which leads to hyperglycemia [1, 2].

The drugs that are currently available in the treatment of diabetes are mainly targeted either to improve insulin sensitivity or improve insulin secretion or both. Even after the discovery and use of insulin and availability of existing modern antidiabetic agents such as sulphonylureas, biguanides, and incretins, the search of more effective drugs of plant origin for the treatment of diabetes continues as an alternative [3–6]. Several medicinal plant parts have demonstrated promising results in terms of achieving normoglycemia by improving insulin secretion from pancreatic beta cells, while some have shown to increase peripheral utilization of glucose [7, 8] or improve hepatic insulin sensitivity [9–11].

*Tinospora crispa* (*T*. *crispa*) belongs to the *Menispermaceae* plant family and is known by various vernacular names such as “*Akar patawali*” or “*Akar seruntum*” (Malays). It comprises a climbing vine found throughout the southwestern part of China to southeast Asia including Malaysia. The aqueous extract of *T. crispa* is used in traditional medicine for treatment of type 2 diabetes [12, 13]. The antidiabetic effects of *T. crispa* extract have been previously demonstrated both *in vivo *and *in vitro *[12–16]. In this study, we have investigated the antidiabetic effect of borapetol B (C1), a compound isolated from *T. crispa* by evaluating the blood glucose levels and stimulation of insulin secretion in normoglycemic control Wistar (W) and diabetic Goto-Kakizaki (GK) rats, an animal model of type 2 diabetes [17].

## 2. Materials and Methods

### 2.1. Animals

Male normoglycemic control Wistar (W) and spontaneously type 2 diabetic Goto-Kakizaki (GK) rats (200–350 g) were used in this study. GK rats, originating from W rats, were bred in our department [17]. W rats were purchased from a commercial breeder (Charles River). The animals were kept at 22°C with an alternating 12-hour light-dark cycle (6 am–6 pm) and were allowed access to food and water before being anesthetized for isolation of pancreatic islets. The study was approved by the Laboratory Animal Ethics Committee of the Karolinska Institutet.

### 2.2. Plant Material

*T. crispa* vines were collected in Kota Belud (Sabah, Malaysia) in May 2005, identified by Berhaman Ahmad (Universiti Malaysia Sabah) and voucher specimen (FRI54832) deposited at the Forest Research Institute Malaysia. The stems were cleaned, air dried (3 days), and ground into coarse powder. Stem powder was sealed and stored at 4°C in a dry cabinet. 

### 2.3. Bioassay-Guided Isolation of Borapetol B

Isolation and purification of borapetol B (C1) from *T. crispa* were modified from a previous study [18]. During the isolation procedure, fractions stimulating insulin secretion in a bioassay with isolated pancreatic islets from W rats were selected for subsequent purification [17].

The stem powder (5 kg) was extracted by sonicating with solvents at room temperature (25°C) for 15 minutes. It was first defatted with hexane (20 L) followed by methanol-water (4 : 1 by volume, 20 L) solvent extraction. Each extraction was repeated 3 times. Extracts were consolidated and reduced to one-third volume by vacuum evaporation yielding a brown syrup. The syrup was acidified to pH 2 with sulphuric acid (50% v/v) and partitioned four times with chloroform. The chloroform layer was evaporated to dryness to obtain brownish mass which showed prominent insulin stimulatory effect. 

The brown mass was chromatographed over normal phase silica gel eluted with 100% chloroform followed by chloroform methanol (9.5 : 0.5) and subsequently increasing eluent polarity with methanol. Chromatographic fractions were monitored by thin layer chromatography (TLC) visualized at 365 nm. Fractions containing spots possessing *R*_*f*_ value within the range of 0.20–0.75 (chloroform methanol 9.5 : 0.5) were examined further as these fractions were also inducing insulin secretion.

**Figure 1:**
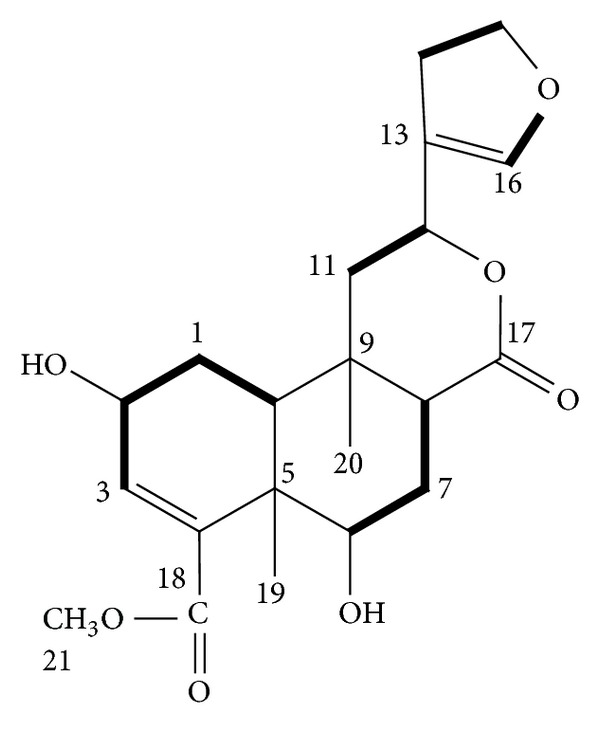
Chemical structure of C1 or borapetol B isolated from *T. crispa.*

These fractions were consolidated and re-chromatographed to yield 10 subfractions. When cooled (4°C), subfractions seven and eight yielded colourless, monoclinic crystals. Upon recrystallization with chloroform-methanol, crystals (450 mg) were recovered by vacuum filtration and washed with cold chloroform. TLC revealed a single compound known as C1 (Figure 1) which stimulated insulin secretion. The identity of C1 as borapetol B was further confirmed using ^1^H-NMR [18, 19]. 

### 2.4. Study Design

**Figure 2:**
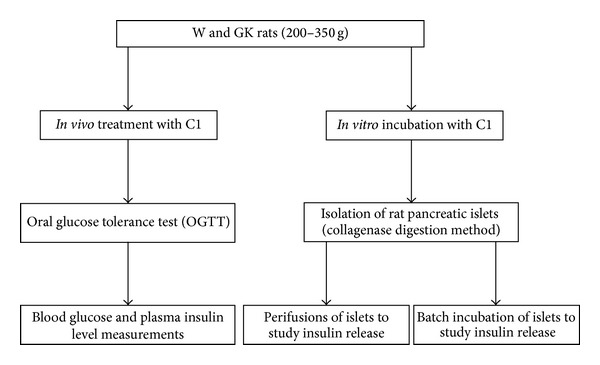
Study design. The *in vivo* and *in vitro* studies were carried out to identify the effects of *T. crispa* C1 in W and GK rats.

The protocol of this study is presented in Figure 2. The effect of C1 on the blood glucose and plasma insulin was assessed by an oral glucose tolerance test in normoglycemic control W and GK rats. For *in vitro *studies, the stimulation of insulin secretion was assessed by performing batch incubation and perifusion experiments using isolated W and GK pancreatic islets.

#### 2.4.1. Oral Glucose Tolerance Test (OGTT)

An OGTT was performed to identify the effect of C1 on the blood glucose levels in W and GK rats. The same rats (*n* = 5) were used for both control (placebo) and treatment groups with 7 days between each type of treatment. The rats were fasted overnight (14-15 hours), allowing access only to plain drinking water. For the treatment group, 10 *μ*g/100 g body weight of C1 was administrated orally by gavage 30 min prior to an oral glucose challenge (0.2 g/100 g body weight). Control rats were given water only. Blood for glucose determination was measured by tail-prick method at different time points: −30 min (before the administration of the compound), 0 min (before glucose load), then at 30, 60, and 120 min after glucose administration. Blood glucose level was measured using a glucometer, Accu-check Aviva (Roche Diagnostic GmbH, USA). Blood samples were also collected for the measurement of plasma insulin levels (about 20 *μ*L/serum sample) at 0 and 30 min. 

#### 2.4.2. Isolation of Pancreatic Islets

Islets from W and GK rats were used for *in vitro* experiments. The isolation of islets was performed using collagenase digestion method. Hank's balanced salt solution (HBBS) (Statensveterinäranstalt, Sweden) containing collagenase (Sigma-Aldrich, USA) was injected through the bile duct. For W and GK rats, 9 mg and 24 mg of collagenase were added to 10 mL of HBBS, respectively. The pancreas was then collected, incubated in 37°C water bath without shaking for 24 min, followed by several washing and centrifugation steps with HBBS, Histopaque 1119 (Sigma-Aldrich, USA), and Histopaque 1077 (Sigma-Aldrich, USA). The islets were hand-picked under a stereomicroscope and then cultured for 24 hours at 37°C, with an atmosphere of  5% CO_2_-95% air in RPMI 1640 culture medium (SVA, Sweden) supplemented with 30 mg L-glutamine (Sigma-Aldrich, USA), 11 mM glucose (Sigma-Aldrich, USA), and antibiotics (100 IU/mL penicillin and 0.1 mg/mL streptomycin) (Invitrogen, USA). Heat-inactivated fetal calf serum (10%) was added to RPMI 1640 medium before the incubation of islets [17].

#### 2.4.3. Batch Incubations for Insulin Secretion

The medium used was Krebs-Ringer bicarbonate (KRB) buffer solution [20] containing 10 mmol/L *HEPES *(Sigma-Aldrich, USA) and 0.2% bovine serum albumin. Following overnight incubation, the islets were preincubated at 3.3 mM glucose for 30–45 min at 37°C with an atmosphere of  5% CO_2_-95% air. After washing islets twice with the incubation medium, batches of 3 islets of similar size were incubated at 3.3 mM or 16.7 mM glucose with or without C1 compound at different concentrations (0.1, 1, and 10 *μ*g/mL). The tubes containing islets and solutions were then incubated for 60 min in 37°C waterbath, slowly shaking. After incubation, 200 *μ*L of the solutions was transferred to new tubes for RIA and kept in freezer −20°C until being assayed for insulin by RIA.

#### 2.4.4. Perifusions of Islets

Perifusions of islets were done to investigate how C1 affects the kinetics of insulin release [20]. Batches of 30 or 50 isolated W and GK rat islets each were layered between polystyrene beads (Bio-Rad Laboratories, Inc., USA) in a perifusion chamber and perifused by use of peristaltic pump (Ismatec SA, Zurich, Switzerland) as previously described [21]. Perifusion medium was collected in fractions every 2 min to establish the basal insulin secretion rate at 3.3 mM glucose for 20 min (−20 to min 0). At min 0 to 15, the glucose concentration was maintained at 3.3 mM glucose and increased to 16.7 mM glucose at 15 to 30 min. Finally, the glucose concentration was then switched back to 3.3 mM glucose. C1 (10 *μ*g/mL) was added at min 0–15 in 3.3 mM glucose and at min 15–30 in 16.7 mM glucose. The fractions were collected and stored in −20°C for insulin radioimmunoassay (RIA).

### 2.5. Insulin RIA

Aliquots obtained from batch incubations and perifusions experiments were analyzed for insulin content using RIA [22].

### 2.6. Statistical Analysis

The results are presented as mean ± SEM. Difference between experimental groups was analyzed using paired *t*-test for OGTT and insulin secretion experiments whereas 2-way ANOVA was used for perifusions of islet experiment. *P* value of less than 0.05 was considered to be significant. All data were analyzed using Prism Graph Pad Software (CA, USA).

## 3. Results

### 3.1. Oral Glucose Tolerance Test (OGTT) in W and GK Rats

**Figure 3:**
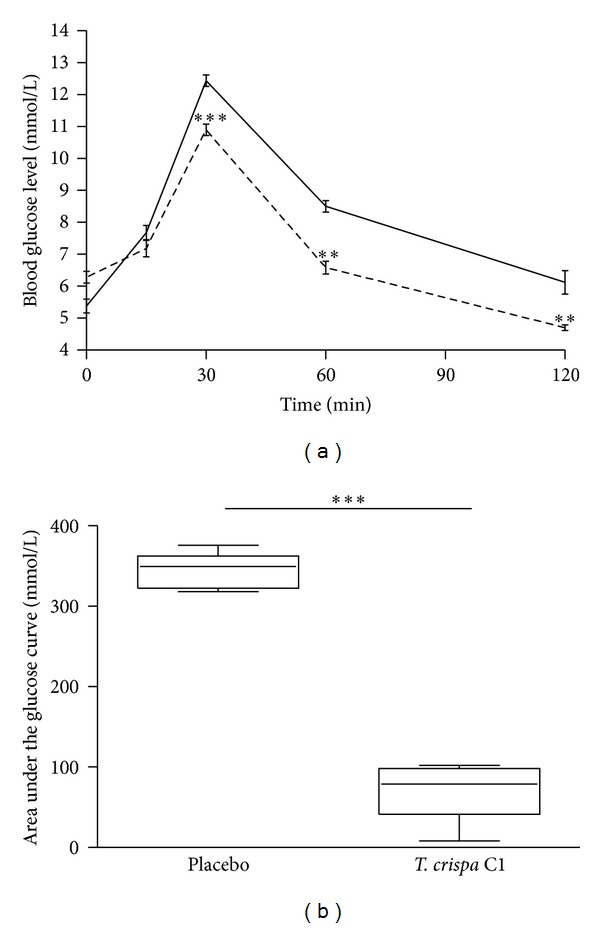
(a) Blood glucose level in the oral glucose tolerance test in W rats. 10 *μ*g/100 g of b.w. of *T. crispa *C1 (- - -) or placebo (—) was given orally 30 minutes prior to the glucose challenge (0.2 g/100 g of b.w.). Data are presented as means ± SEM (*n* = 5). **P < 0.01 versus placebo; ***P < 0.001 versus placebo. (b) Area under the glucose curve in the oral glucose tolerance test in W rats. Data are presented as means ± SEM (*n* = 5). ***P < 0.001 versus placebo

**Figure 4:**
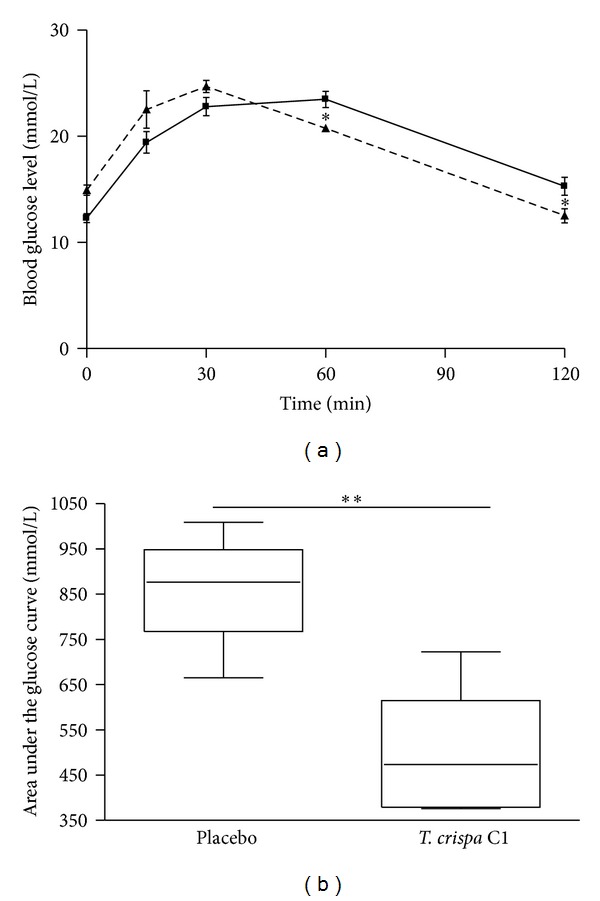
(a) Blood glucose level in the oral glucose tolerance test in GK rats. 10 *μ*g/100 g of b.w of *T. crispa *C1 (- - -) or placebo (—) was given orally 30 minutes prior to the glucose challenge (0.2 g/100 g of b.w.). Data are presented as means ± SEM (*n* = 5). *P < 0.05 versus placebo. (b) Area under the glucose curve in the oral glucose tolerance test in GK rats. Data are presented as means ± SEM (*n* = 5). **P < 0.01 versus placebo.

**Figure 5:**
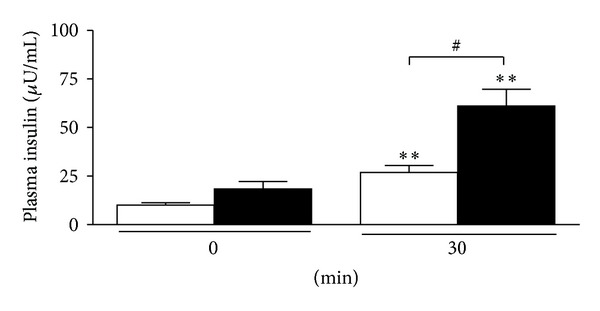
Plasma insulin level at 0 and 30 min in the oral glucose tolerance test from W rats. Data are presented as means ± SEM (*n* = 5). *T. crispa *C1 (■) or placebo (□). **P < 0.01 versus 0 min (placebo and *T. crispa *C1, resp.); ^#^ *P* < 0.05 versus 30 min (placebo).

**Figure 6:**
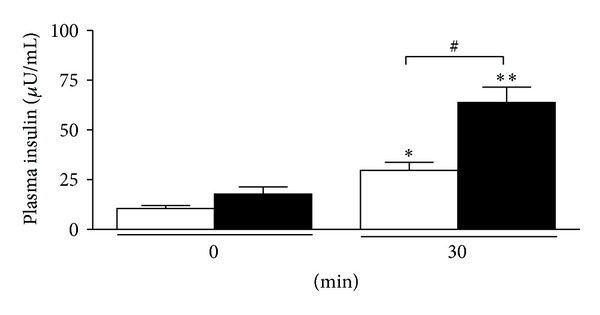
Plasma insulin level at 0 and 30 min in the oral glucose tolerance test from GK rats. Data are presented as means ± SEM (*n* = 5). *T. crispa *C1 (■) or placebo (□).*P < 0.05 versus 0 min (placebo); **P < 0.01 versus 0 min (*T. crispa *C1); ^#^ *P* < 0.05 versus 30 min (placebo).

After the oral administration of glucose, the blood glucose levels reached a peak at 30 minutes and then gradually decreased in both W and GK rats. In W rats, the blood glucose levels at 30, 60, and 120 min were significantly decreased in the treated group as compared to the placebo group (at 120 min 4.7 ± 0.1 versus 6.1 ± 0.4 to mmol/L; *P* < 0.01) (Figure 3(a)) and with areas under the glucose curves (AUCs) (0 min to 120 min) being 72 ± 17 versus 344 ± 10 mmol/L (*P* < 0.001) (Figure 3(b)). In GK rats, the blood glucose levels at 60 and 120 min were significantly decreased in the treated group as compared to the placebo group (at 120 min 12.5 ± 0.8 versus 15.3 ± 0.9  mmol/L; *P* < 0.05) (Figure 4(a)) with AUCs (0–120 min) being 492 ± 63 versus 862 ± 55 mmol/L (*P* < 0.01) (Figure 4(b)). In W and GK rats, plasma insulin levels were increased from 0 min to 30 min in both placebo and treated groups (Figures 5 and 6) and there was a significant difference observed between the placebo and treated groups at 30 min (*P* < 0.05). In W rat, the mean values for plasma insulin in the treated and placebo groups at 0 min were 18 ± 4 *μ*U/mL versus 10 ± 1 *μ*U/mL respectively and at 30 min, 61 ± 9 *μ*U/mL versus 27 ± 4 *μ*U/mL (*P* < 0.05), respectively (Figure 5). In GK, the mean values for plasma insulin in the treated and placebo groups at 0 min were 18 ± 4 *μ*U/mL versus 11 ± 1 *μ*U/mL and at 30 min, 64 ± 8 *μ*U/mL versus 30 ± 4 *μ*U/mL (*P* < 0.05), respectively (Figure 6).

### 3.2. Effects of C1 on Insulin Secretion of W Rat Islets and GK Rat Islets

In W rat islets, the incubation of islets with C1 at 0.1, 1, and 10 *μ*g/mL in 3.3 mM glucose significantly increased the insulin secretion 6.3-fold (*P* < 0.01), 8.1-fold (*P* < 0.05) and 9.1-fold (*P* < 0.001), respectively, compared to the control group (Table 1). At 16.7 mM glucose concentration, C1 (0.1, 1, and 10 *μ*g/mL) stimulated insulin secretion 1.5-fold (*P* < 0.05), 1.9-fold (*P* < 0.05), and 5.0-fold (*P* < 0.001), respectively, compared to the control group. The incubation of GK rat islets with C1 at 0.1, 1, and 10 *μ*g/mL in 3.3 mM glucose significantly increased the insulin secretion 3.9-fold, 6.3-fold and 8.8-fold (all *P* < 0.05), respectively, compared to the control group. At 16.7 mM glucose, C1 at 0.1, 1, and 10 *μ*g/mL stimulated insulin release 1.5-fold, 2.3-fold, and 4.2-fold (all *P* < 0.05), respectively, compared to the control group.

### 3.3. Kinetics of Insulin Secretion of Isolated Islets

**Figure 7:**
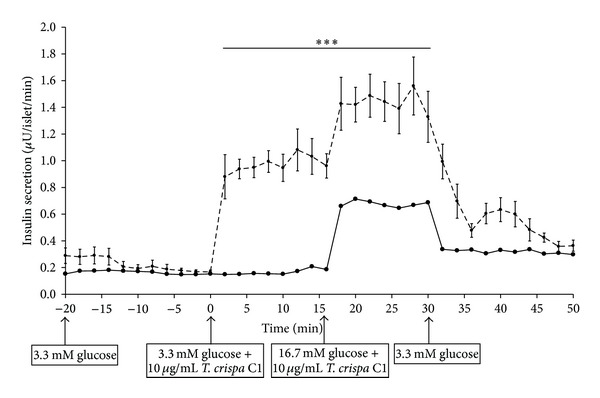
Effect of *T. crispa *C1 on kinetics of insulin secretion of W rat islets. Data are presented as means ± SEM from six separate experiments. Aliquots of the medium were collected and then determined by RIA. *T. crispa *C1 (- - -) or control (—). ***P < 0.001 versus control.

**Figure 8:**
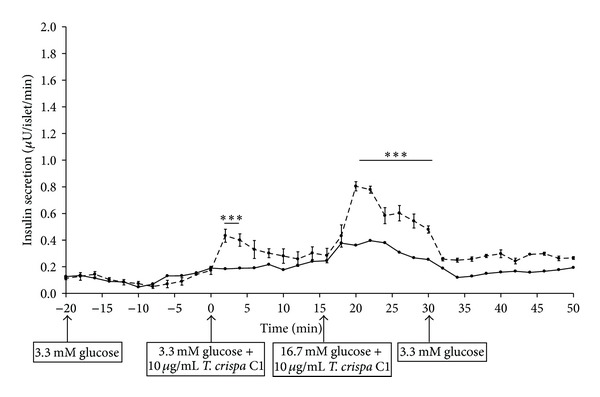
Effect of *T. crispa *C1 on kinetics of insulin secretion of GK rat islets. Data are presented as means ± SEM from three separate experiments. Aliquots of the medium were collected and then determined by RIA. *T. crispa *C1 (- - -) or control (—). ***P < 0.001 versus control.

Insulin secretion was increased as 10 *μ*g/mL of C1 was added to perifusate containing 3.3 mM (0–16 min) and 16.7 mM (16–30 min) glucose in both W (Figure 7) and GK (Figure 8) rat islets. In W, the addition of C1 stimulated insulin secretion by 5.5-fold from 0.2 ± 0.01 *μ*U/islet/min (0 min) to 1.1 ± 0.16 *μ*U/islet/min (12 min) and further increased to 7.5-fold (1.5 ± 0.16 *μ*U/islet/min) (22 min). There was a significant difference observed from 2 min to 32 min (*P* < 0.001) in the treated group compared to control (Figure 7). In GK rat, C1 stimulated insulin secretion by 2.5-fold from 0.17 ± 0.03 *μ*U/islet/min (0 min) to 0.43 ± 0.05 *μ*U/islet/min (2 min) (*P* < 0.001). The insulin secretion was increased to 0.8 ± 0.03 *μ*U/islet/min (20 min) (*P* < 0.001) in 16.7 mM glucose. There was a significant difference observed between the treated and control group at 2 to 4 min (*P* < 0.001) and between 20 to 30 min (*P* < 0.001) (Figure 8). The insulin secretion returned to basal level in both W and GK when C1 was omitted from the perifusate.

## 4. Discussion

We show that oral administration of *T. crispa* C1 30 minute before an oral glucose challenge significantly decreased blood glucose levels in W rats. This was most likely mediated through enhanced insulin secretion, since the plasma insulin level in treated W rats increased by 2-fold compared to the placebo group. This is further supported by our findings that C1 stimulates insulin secretion from isolated pancreatic islets, both in batch incubations and in perifusions.

A major defect behind type 2 diabetes is inadequate insulin secretion, that would be needed to compensate for decreased insulin sensitivity [1, 2]. This in turn leads to development of hyperglycemia. To further assess the insulinotropic properties of C1 in type 2 diabetes, studies were performed in spontaneously diabetic Goto-Kakizaki (GK) rats. The GK rat strain was established from normoglycemic W rats by repeated inbreeding in each successive generation of the siblings with the highest blood glucose levels during an OGTT [23]. GK rats are lean and develop mild hyperglycemia early in life due to impaired insulin secretion, in particular a consistently low insulin response to glucose stimulation [17, 24]. Impaired glucose-stimulated insulin secretion has been demonstrated *in vivo*, in the perifused isolated pancreas and in isolated pancreatic islets of GK rats [24].

We now demonstrated that oral treatment with C1 decreased blood glucose levels in parallel with an increase in plasma insulin levels during the OGTT, not only in W rats but also in GK rats. In addition, C1 at different concentrations increased insulin release from GK rat islets in low and high glucose and the stimulatory effect was observed in a dose-dependent manner. In the perifusions of islets experiment, C1 stimulated insulin secretion in both W and GK rat islets. The insulin secretion gradually returned to basal level on the removal of C1, supporting that C1 did not cause nonspecific insulin leakage by damaging islets beta cells. 

Previous findings have shown the effectiveness of extract and isolated compounds from *T. crispa* in the stimulation of insulin release and insulin sensitivity in normal and diabetic animal models [12, 14]. A two-week treatment with *T. crispa* extract significantly reduced the blood glucose level and caused a significant increase in plasma insulin in moderately diabetic rats with some functional *β*-cells. No effect was observed in severely diabetic animals suggesting that the hypoglycemic effect of *T. crispa *extract was not due to extra pancreatic action but through the stimulation of insulin secretion [13].

An *in vitro* study showed that *T. crispa *extract induced a dosage-dependent stimulation and also potentiated basal and glucose-stimulated secretion of insulin in rat islets and HIT-T15 cells. The insulin secretion in perifused isolated human and rat islets as well as HIT-T15 *β* cells returned to basal levels as *T. crispa *extract was omitted from perifusate indicating that the insulintropic activity was not due to toxicity effect [14]. 

Another compound isolated from *T. crispa *borapetoside C reduced plasma glucose levels in normal and type 2 diabetic mice and streptozotocin induced type 1 diabetic mice but increased plasma insulin levels in normal and type 2 diabetic mice only. The hypoglycemic effect was associated with increase of glucose utilization in peripheral tissues and the reduction of hepatic gluconeogenesis [15]. In another study, borapetoside C increased glucose utilization, delayed the development of insulin resistance, and enhanced insulin sensitivity in diabetic mice [16]. 

It would have been interesting to explore the anti-diabetic effects of borapetoside C with borapetol B (C1) and to compare with the effects of C1 in W and GK rats. Since GK rats in addition to defective insulin secretion display decreased insulin sensitivity, it is possible that a stronger antidiabetic effect would be obtained by treatment with a combination of C1 and borapetoside C. However, due to limitations and difficulties to obtain borapetoside B, we have focused on the effects of C1.

## 5. Conclusion

We demonstrate that *T. crispa *C1 improves the diabetic condition in GK rats by stimulating insulin secretion. Further studies are needed to understand the mechanisms involved by which *T. crispa *C1 induces insulin release from pancreatic islets.

## Figures and Tables

**Table 1:** The effect of different concentrations of *T. crispa *C1 at low (3.3 mM) and high (16.7 mM) glucose on insulin secretion from W (*n* = 5) and GK (*n* = 3) rat islets.

Addition to incubation medium		Insulin release (*μ*U/islet/h)	
Glucose (mM)	*T. crispa* C1 (*μ*g/mL)	W islets	GK islets
3.3	None	2.4 ± 0.1	1.2 ± 0.2
	0.1	15.1 ± 2.2**	4.7 ± 0.3*
	1	19.5 ± 4*	7.5 ± 0.9*
	10	21.9 ± 2***	10.5 ± 1.8*

16.7	None	32.5 ± 1.8	13.6 ± 0.6
	0.1	48.8 ± 6^#^	20.6 ± 1.1^#^
	1	63.1 ± 8.9^#^	30.9 ± 3.8^#^
	10	164.5 ± 11.3^###^	57.3 ± 6.3^#^

Data are presented as means ± SEM. W: *P < 0.05 versus 3.3 mM glucose; **P < 0.01 versus 3.3 mM glucose; ***P < 0.001 versus 3.3 mM glucose; ^#^ *P* < 0.05 versus 16.7 mM glucose; ^###^ *P* < 0.001 versus 16.7 mM glucose GK: *P < 0.05 versus 3.3 mM glucose; ^#^ *P* < 0.05 versus 16.7 mM glucose.

## References

[1] Schofield CJ, Sutherland C (2012). Disordered insulin secretion in the development of insulin resistance and type 2 diabetes. *Diabetes Medicine*.

[2] Lin Y, Sun Z (2010). Current views on type 2 diabetes. *Journal of Endocrinology*.

[3] Lin CC (1992). Crude drugs used for the treatment of diabetes mellitus in Taiwan. *American Journal of Chinese Medicine*.

[4] Famuyiwa OO (1993). The efficacy of traditional medicine in the management of diabetes mellitus in southwestern Nigeria. *African Journal of Medicine and Medical Sciences*.

[5] Baker JT, Borris RP, Carté B (1995). Natural product drug discovery and development: new perspectives on international collaboration. *Journal of Natural Products*.

[6] Yeh GY, Eisenberg DM, Kaptchuk TJ, Phillips RS (2003). Systematic review of herbs and dietary supplements for glycemic control in diabetes. *Diabetes Care*.

[7] Kavishankar GB, Lakshmidevi N, Mahadeva Murthy S (2011). Phytochemical alysis ad atimicrobial properties of selected medicinal plats against bacteria associated with diabetic patients. *International Journal of Pharma and Bio Sciences*.

[8] Patel DK, Prasad SK, Kumar R, Hemalatha S (2012). An overview on antidiabetic medicinal plants having insulin mimetic property. *Asian Pacific Journal of Tropical Biomedicine*.

[9] Yassin K, Huyen VTT, Hoa KN, Östenson CG (2011). Herbal extract of *Gynostemma pentaphyllum* decreases hepatic glucose output in type 2 diabetic goto-kakizaki rats. *International Journal of Biomedical Science*.

[10] Huyen VTT, Phan DV, Thang P, Ky PT, Hoa NK, Östenson CG (2012). Antidiabetic effects of add-on *Gynostemma Pentaphyllum* extract therapy with sulfonylureas in type 2 diabetic patients. *Evidence-Based Complementary and Alternative Medicine*.

[11] Huyen VTT, Phan DV, Thang P, Hoa NK, Östenson CG (2013). *Gynostemma pentaphyllum* tea improves insulin sensitivity in type 2 diabetic patients. *Journal of Nutrition Metabolism*.

[12] Noor H, Ashcroft SJH (1989). Antidiabetic effects of *Tinospora crispa* in rats. *Journal of Ethnopharmacology*.

[13] Noor H, Ashcroft SJH (1998). Pharmacological characterisation of the antihyperglycaemic properties of *Tinospora crispa* extract. *Journal of Ethnopharmacology*.

[14] Noor H, Hammonds P, Sutton R, Ashcroft SJH (1989). The hypoglycaemic and insulinotropic activity of *Tinospora crispa*: studies with human and rat islets and HIT-T15 B cells. *Diabetologia*.

[15] Lam S-H, Ruan C-T, Hsieh P-H, Su M-J, Lee S-S (2012). Hypoglycemic diterpenoids from *Tinospora crispa*. *Journal of Natural Products*.

[16] Ruan C-T, Lam S-H, Chi T-C, Lee S-S, Su M-J (2012). Borapetoside C from *Tinospora crispa* improves insulin sensitivity in diabetic mice. *Phytomedicine*.

[17] Östenson C-G, Khan A, Abdel-Halim SM (1993). Abnormalin insulin secretion and glucose metabolism in pancreatic islets from the spontaneously diabetic GK rat. *Diabetologia*.

[18] Fukuda N, Yonemitsu M, Kimura T (1986). Studies on the constituents of the stems of *Tinospora tuberculata* Beumee. III. New diterpenoids, Borapetoside B and Borapetol B. *Chemical and Pharmaceutical Buletin*.

[19] Choudhary MI, Ismail M, Shaari K (2010). *Cis*- clerodane-type furanoditerpenoids from tinospora crispa. *Journal of Natural Products*.

[20] Hoa NK, Norberg Å, Sillard R (2007). The possible mechanisms by which phanoside stimulates insulin secretion from rat islets. *Journal of Endocrinology*.

[21] Bjorklund A, Grill V (1993). B-cell insensitivity in vitro: reversal by diazoxide entails more than one event in stimulus-secretion coupling. *Endocrinology*.

[22] Herbert V, Lau KS, Gottlieb CW, Bleicher SJ (1965). Coated charcoal immunoassay of insulin. *Journal of Clinical Endocrinology and Metabolism*.

[23] Goto Y, Kakizaki M, Masaki N (1975). Spontaneous diabetes produced by selective breeding of normal Wistar rats. *Proceedings of the Japan Academy*.

[24] Östenson C-G, Efendic S (2007). Islet gene expression and function in type 2 diabetes; studies in the Goto-Kakizaki rat and humans. *Diabetes, Obesity and Metabolism*.

